# Changes in toxin production of environmental *Pseudomonas aeruginosa* isolates exposed to sub-inhibitory concentrations of three common antibiotics

**DOI:** 10.1371/journal.pone.0248014

**Published:** 2021-03-04

**Authors:** Biljana Mojsoska, Melanie Ghoul, Gabriel G. Perron, Håvard Jenssen, Fatima AlZahra’a Alatraktchi

**Affiliations:** 1 Department of Science and Environment, Roskilde University, Roskilde, Denmark; 2 PreDiagnose, Karlslunde, Denmark; 3 Department of Zoology, University of Oxford, Oxford, United Kingdom; 4 Department of Biology, Bard College, Annandale-On-Hudson, NY, United States of America; Cornell University, UNITED STATES

## Abstract

Pseudomonas aeruginosa is an environmental pathogen that can cause severe infections in immunocompromised patients. *P*. *aeruginosa* infections are typically treated with multiple antibiotics including tobramycin, ciprofloxacin, and meropenem. However, antibiotics do not always entirely clear the bacteria from the infection site, where they may remain virulent. This is because the effective antibiotic concentration and diffusion in vitro may differ from the in vivo environment in patients. Therefore, it is important to understand the effect of non-lethal sub-inhibitory antibiotic concentrations on bacterial phenotype. Here, we investigate if sub-inhibitory antimicrobial concentrations cause alterations in bacterial virulence factor production using pyocyanin as a model toxin. We tested this using the aforementioned antibiotics on 10 environmental *P*. *aeruginosa* strains. Using on-the-spot electrochemical screening, we were able to directly quantify changes in production of pyocyanin in a measurement time of 17 seconds. Upon selecting 3 representative strains to further test the effects of sub-minimum inhibitory concentration (MICs), we found that pyocyanin production changed significantly when the bacteria were exposed to 10-fold MIC of the 3 antibiotics tested, and this was strain specific. A series of biologically relevant measured pyocyanin concentrations were also used to assess the effects of increased virulence on a culture of epithelial cells. We found a decreased viability of the epithelial cells when incubated with biologically relevant pyocyanin concentrations. This suggests that the antibiotic-induced virulence also is a value worth being enclosed in regular testing of pathogens.

## Introduction

The rapid emergence of antibiotic resistance is endangering the efficacy of antibiotics [1]. The susceptibility profile of bacterial pathogens provides valuable information to prescribe the most efficient antibiotic treatment. While it is known that the antimicrobial impact indeed is not an ‘all or none’ effect, treatment without antimicrobial susceptibility information may contribute to recurring and chronic infections [2–4]. The effect of sub-inhibitory antimicrobial concentrations on bacterial virulence is a field that is not intensively studied. This calls for increased efforts to understand the implication of sub-inhibitory concentrations of antimicrobials on bacterial virulence profiles.

Pseudomonas aeruginosa is an opportunistic pathogen listed by the World Health Organization as one of the three pathogens that causes severe infections and high mortality in hospital patients [5–7]. Current antibiotic treatment for *P*. *aeruginosa* infections are classically placed amongst the fluoroquinolones (e.g. ciprofloxacin), the beta-lactams (e.g. meropenem), and the aminoglycosides (e.g. tobramycin, gentamicin), with polymyxins being used as a last resort option [8,9]. Antibiotic treatment has inevitably led to evolution of many antibiotic-resistant mechanisms within this pathogen, making it difficult to treat in the clinic.

*P*. *aeruginosa* is known for its ability to produce numerous virulence factors that play an important role in the early phases of bacterial host colonization [10]. One of these virulence factors is pyocyanin, a quorum-sensing regulated toxin that plays an important role in killing host epithelial cells, and has an antagonistic effect towards other bacterial species [11,12]. Pyocyanin has been studied concerning human infections; however, its role and secretion pattern in environmental strains of *P*. *aeruginosa* has not been investigated. The toxicity exerted by pyocyanin is due to their ability to enter the cell interior and use oxygen as an electron acceptor subsequently generating reactive oxygen species (ROS) (e.g. hydrogen peroxide) and induce oxidative stress to the cell. Studies have shown that pyocyanin exerts minor toxic events in the range of 5–10 mg/L in lung epithelial cells (L-132) [13]. Additionally, Saunders et al. suggest that pyocyanin plays an important role in the metabolism of *P*. *aeruginosa* biofilm due to its participation in charge transfer processes [14].

Traditionally, virulence molecules are determined by extensive laboratory processing, including chemical and mechanical sample pretreatment, chemical extraction and often complicated and time-consuming signal identification procedures [15]. Such laboratory-intensive protocols often lack precision and are difficult to conduct for high-throughput screenings [4]. For example, pre-treatment often leads to loss of sample quality during sample preparation steps. Therefore, new technologies for rapid, high-throughput and sensitive screening of virulence factors are needed. Direct on-the-spot screening of pyocyanin in un-treated samples using electrochemical sensors is an emerging field that is gaining increased attention for its simplicity and rapid quantification [16–19].

Here, we utilize the high-throughput electrochemical method that does not require sample preparation and allows for the identification and quantification of pyocyanin in 17 seconds. We screened 10 different *P*. *aeruginosa* environmental strains and the laboratory strain PAO1 for their pyocyanin activity in response to treatment with antibiotics from the three core classes, i.e. ciprofloxacin, meropenem, and tobramycin at varying concentrations. The quantification of pyocyanin enabled by this technique revealed that sub-inhibitory antibiotic concentrations induce increased pyocyanin secretion. Crucially, the increased pyocyanin production is in a range that has been reported to be detected in patients with *P*. *aeruginosa* infections [12,20,21]. Finally, we found that this increase in pyocyanin production has an antagonistic effect on epithelial cells. These findings suggest that patients receiving antibiotic treatment could experience side effects such as worsened pathogenic virulence. Similarly, due to the effect of antibiotic pollution in the environment [22], our results suggest that environmental strains of *P*. *aeruginosa*, an important source of infection in critical care units and cystic fibrosis patients [23] could also experience increased virulence.

## Materials and methods

### Strain collection

The *Pseudomonas aeruginosa* (Pae) strains used in the present study are environmental isolates from soil, sediment, and water (S1 Table) which have previously been published [24]. PAO1 was used as a reference strain.

### Determining the minimum inhibitory concentrations

Minimum inhibitory concentrations (MIC) for ciprofloxacin, tobramycin and meropenem were obtained using E-test strip diffusion and micro broth dilution methods. E-test plastic strips (Liofilchem, Italy) that have a predefined gradient of antibiotic concentration [25]. A 0.5 McFarland standard suspension was inoculated onto Mueller-Hinton agar plates. Up to four E-test strips were placed on each plate following the manufacturer’s instructions (Liofilchem, Italy). The concentrations measured were: ciprofloxacin: 0.002–32 μg/mL, tobramycin: 0.016–256 μg/mL, and meropenem: 0.002–32 μg/mL. For the micro broth dilution method, we diluted overnight cultures of *P*. *aeruginosa* isolates (1:50) in fresh LB media, which we allowed to grow to exponential phase (OD600 = 0.4) and then diluted to 2–8 10^5^ colony forming units (CFU) per mL in 96-well polypropylene plates with or without series of concentrations of the three antibiotics tested. The plates were incubated at 37°C and after 20–24 hours the highest concentration where no visible growth was observed was noted as the MIC value. The 10x-sub MIC values used in the present study for the three tested strains were the following: PAO1 (Cipro = 0.0125 μg/mL; Tobra = 0.0125 μg/mL, Mero = 0.05 μg/mL), Pae85 (Cipro = 0.0125 μg/mL; Tobra = 0.1 μg/mL, Mero = 0.025 μg/mL) and Pae112 (Cipro = 0.025 μg/mL; Tobra = 0.05 μg/mL, Mero = 0.025 μg/mL). The applied standardized MIC protocol is based on Wiegand et al. 2008 [26].

### Electrochemical screening of pyocyanin

Pyocyanin is a redox-active molecule possible to detect directly with electrochemical sensors as previously described [17,18]. All measurements were conducted by pipetting 50 μL of the sample on a single-use disposable sensor (GPH110, Metroohm, Denmark) operated by a potentiostat (EmstatBlue, PalmSens, Netherlands) using a square voltammetry procedure measuring from -0.6 V to 0.0 V at a step potential of 0.05 and a total run-time of 17 seconds. First, we generated calibration curves for pyocyanin by measuring three independent serial dilutions of pure pyocyanin (P0046, Merck, Denmark) prepared in lysogeny broth (LB) media ranging between 1.5 μM and 24 μM. To measure the concentrations of pyocyanin produced by the ten environmental bacterial strains and strain PAO1, we grew fresh bacterial cultures in round bottom glass tubes containing 3 ml LB for 24 hours at 37°C and shaking at 180 rpm. Then, we diluted the cultures to starting inoculum OD600 of 0.01 in round bottom glass tubes containing 3 ml LB and allowed them to grow for another 24 hours to assure stationary phase for all different isolates before 50 μL was taken for measurements. The experiments were conducted in five biological replicates.

### Determining the pyocyanin production in presence of antibiotics

Selected environmental strains Pae85 and Pae112 and PAO1 were grown for 24 hours after which the cultures were diluted to an optical density (OD_600_ nm) of 0.01 and allowed to grow until OD _600_ nm of 0.1–0.2 was reached. At this stage, 50 μL samples were taken for measuring the concentration of pyocyanin. A small volume (2 mL) of each of the cultures was taken into 3 separate tubes and antibiotics at concentrations below the MIC was added. The bacteria with and without antibiotics were allowed to grow for 24 hours, at 37°C and shaking at 180 rpm. After the 24 hours of incubation, 50 μL of each of the cultures with and without antibiotics for each of the strains was taken for quantification. The OD_600_ nm for each of the isolates was measured and used for normalization of the production of pyocyanin. To confirm that there was no chemical interference between the antibiotic and the virulence factor that would affect the measurements, samples of a constant concentration of pyocyanin spiked with each of the three antibiotics were measured (Cipro = 0.125 μg/mL; Tobra = 0.25 μg/mL, Mero = 1 μg/mL). Additionally, different concentrations of tobramycin were added to the supernatant of PAO1 and measured electrochemically. The electrochemical measurements of pyocyanin did not change (S1 Fig).

### Determining the effect of virulence factor on epithelial cells

To evaluate the degree of toxicity of pyocyanin production on a potential host, we assessed the cytotoxicity of pyocyanin produced in the supernatant of strain PAO1 on HaCaT cells–human immortalized keratinocytes that are the predominant human cell types of the epidermis [27]. HaCaT cells [28] were provided by Bispebjerg Hospital, Denmark. We obtained PAO1 supernatant from an overnight culture that we centrifuged and filtered so that no viable cells or cell debris was present. Sterility of the supernatant after filter purification was confirmed by plating 100 μL of the supernatant on Pseudomonas isolation agar plates followed by 24 hours incubation at 37°C. Cytotoxicity was determined using an assay previously described [29]. Briefly, the assay relies on the ability of viable cells to convert the novel tetrazolium salt MTS [(3-(4,5-dimethylthiazol-2-yl)-5-(3-carboxymethoxyphenyl)-2-(4-sulfophenyl)-2H-tetrazolium] into a soluble formazan product, and will hereafter be referred to as MTS cytotoxicity assay. Absorbance at 490 nm is measured and is directly proportional to the viability of the cells. Keratinocytes were cultured in Dulbecco’s modified Eagle’s medium (DMEM) with glutamax (Thermofisher, Denmark), supplemented with 100 U/mL penicillin/streptomycin and 10% heat-inactivated fetal bovine serum (FBS GIBCO; Thermofisher, Denmark). Cells were seeded at a concentration of 15 × 10^4^ per well in a sterile, flat-bottom 96-well polystyrene plates for 24 hours and then carefully washed with phosphate-buffered saline (PBS) without Ca^+2^ and Mg^+2^. Pyocyanin (CAS:85-66-5, Merck, Germany) in DMEM at 5 different concentrations and PAO1 supernatant was added to each well. The plates were incubated for 24 hours in a humidified 5% CO_2_ atmosphere at 37°C, after which 20 μL of MTS -phenazine methosulfate (PMS) solution was added, and the plate was further incubated for 2 hours for colour development. Absorbance at 490 nm was measured using the Synergy HT microplate reader. For viability controls and baseline correction, 1% Triton X-100 (0% viability), untreated cells (100% viability), and cell-free DMEM were used. Additionally, pyocyanin and PAO1 supernatant in DMEM at the highest concentration were also measured for the possibility of colour interference. The percent viability was expressed as the concentration of pyocyanin standard and from PAO1 supernatant that reduces cell viability by 50%.

### Statistical analysis

For the statistical analysis of the data, GraphPad Prism version 7.00, GraphPad Software, La Jolla California, USA was used. For the overall pyocyanin production in all the strains tested, we used a one-way ANOVA followed by a Tuckey post hoc test to investigate differences between treatments. For changes in pyocyanin production in presence of antibiotics, we used two statistical comparisons. For multiple comparisons, we calculated adjusted P values (adj-P) using one-way ANOVA with Bonferroni correction and for single treatment analysis, we used a series of paired t-tests.

## Results

### Quantification of pyocyanin produced by environmental strains

**Fig 1:**
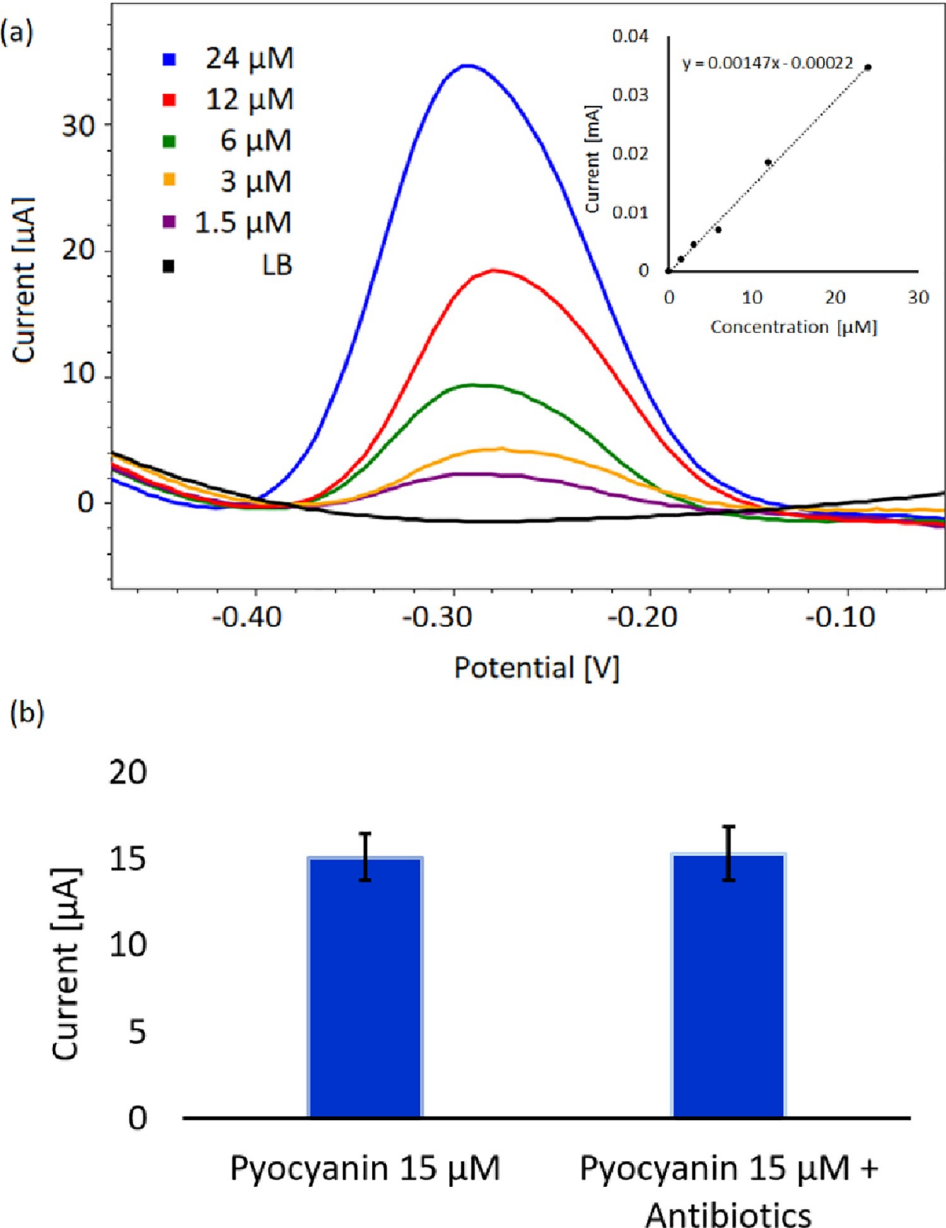
Electrochemical detection of pyocyanin. (a) Square wave voltammogram of pyocyanin in Lysogeny Broth media. Inset shows a standard curve with extracted peak heights from square wave voltammograms as a function of pyocyanin concentration. (b) Electrochemical pyocyanin detection with and without the presence of all three antibiotics in the sample (Means with SD from 3 replicates).

The characteristic electrochemical peak of pyocyanin was found at ~ -0.29 V using square wave voltammetry (SWV) as displayed in Fig 1A. Increasing pyocyanin concentrations result in higher peak heights as denoted by the inset where the concentration is plotted as function of the current. A linear fit of the data reveals linearity of R^2^ = 0.997. The calibration curve was used to convert the current measurements of the culture samples into pyocyanin concentration. A constant pyocyanin concentration was measured with the presence of tobramycin, ciprofloxacin and meropenem, confirming that the antibiotics do not affect the electrochemical signal of pyocyanin (Fig 1B). The potentials at which other relevant molecules undergo redox-reactions do not overlap with the peak potential of pyocyanin as demonstrated by previous electrochemical studies of pyoverdine, 4-hydroxy-2-heptylquinoline *(*HHQ), 2-heptyl-3,4-dihydroxyquinoline *(*PQS), phenazine-1-carboxylic acid and 5-methylphenazine-1-carboxylic acid [16,19,30]. Thus, the detection window of pyocyanin in LB is not in conflict with the detection window of other relevant molecules.

**Fig 2:**
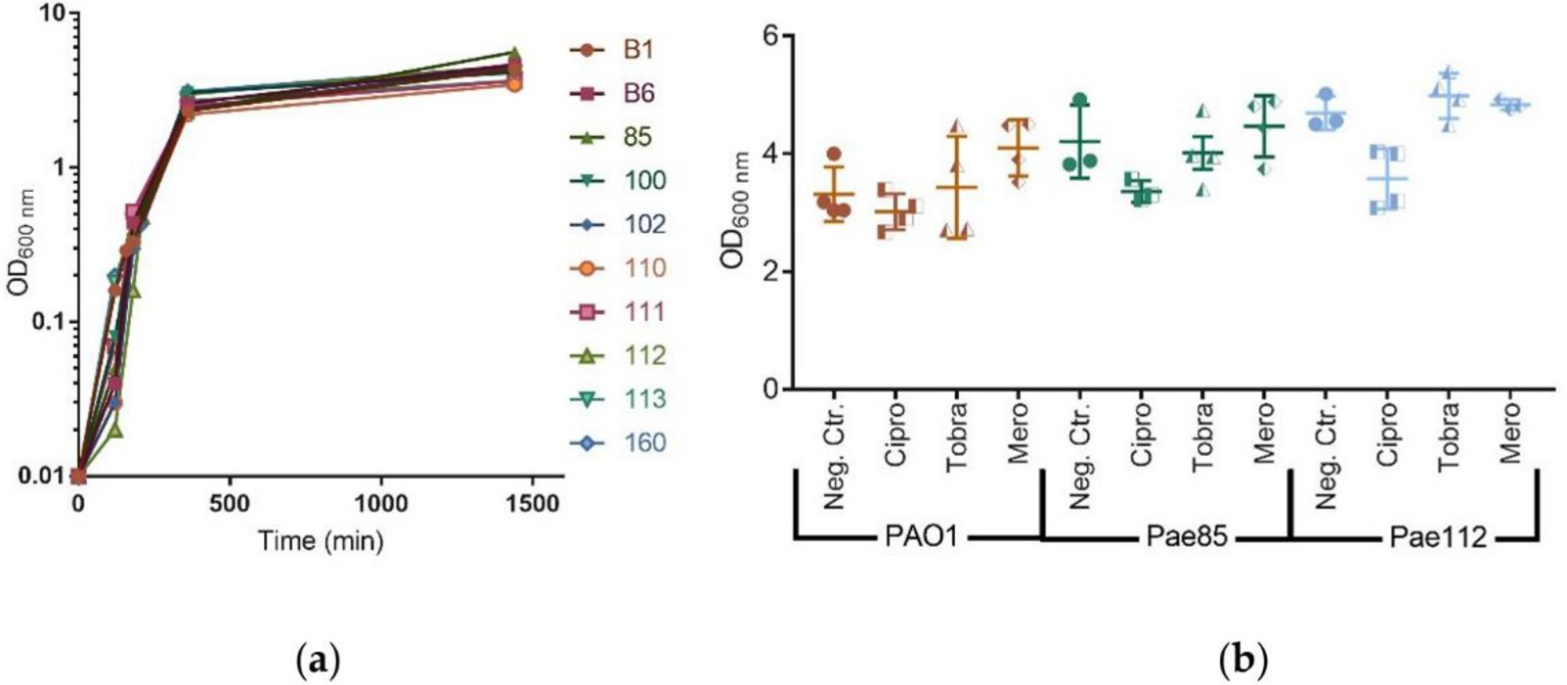
*P*. *aeruginosa* growth curves when exposed to sub-MIC antibiotic concentrations. (a) Growth curves of the 10 environmental strains showing stationary phase after 24 hours of growth under shaking in 37°C in lysogeny broth. (b) Evaluation of the effect of concentrations of ciprofloxacin (Cipro), tobramycin (Tobra), and meropenem (Mero) at 10x sub-MIC values on the growth of strains. Statistical analysis revealed non-significant changes in presence of low antibiotic concentrations for the tested strains (paired t-test, Ps>0.05). Means ± SEM are presented and estimated from 4 replicates.

**Fig 3:**
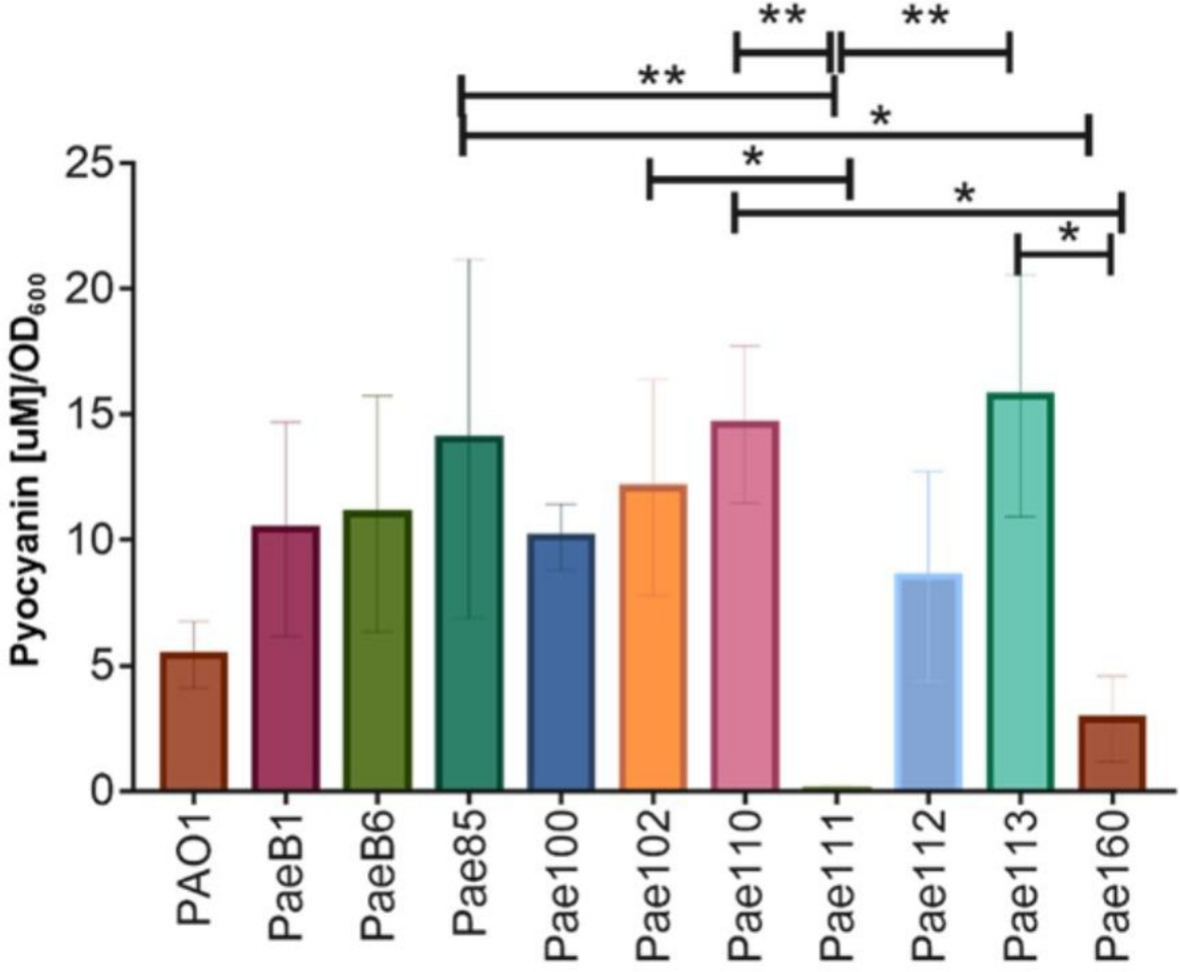
Quantification of production of pyocyanin in environmental strains of *P*. *aeruginosa* and PAO1. *P*. *aeruginosa* strains were grown in LB media at 37°C, 180 rpm for 24 hours until stationary phase was reached. One-way ANOVA multiple comparisons with Tuckey post hoc test (*Ps<0.05, **Ps<0.007) reveals a significant difference in pyocyanin production between the strains. Data showing normalization using maximum pyocyanin production in 10 environmental strains of *P*. *aeruginosa* and PAO1 (*F*_(10,25)_ = 4.799, *P* = 0.0007). Data normalized using optical density (OD_600_nm), means ± SD are presented and estimated from a minimum of three replicates.

The level of heterogeneity in pyocyanin production was investigated using electrochemical measurements. The pyocyanin was measured after 24 hours to assure stationary phase for all different isolates as seen in Fig 2A. The effect of 10× sub-MIC concentrations of the three antibiotics on the growth of the three bacteria strains was examined (Fig 2B). Overall, we found that the three antibiotics did not affect growth for any of the strains when compared to growth in the absence of the antibiotics (Paired t-tests; *Ps* > 0.05; S2 Table). This result enabled us to compare pyocyanin production directly between the different treatments. Different strains of the bacteria are expected to produce different levels of exopolysaccharides and secreted proteins, influencing turbidity, viscosity and growth rate of the individual strains. Therefore, we corrected the pyocyanin concentrations for optical density (OD_600_ nm) (Fig 3). We found that eight out of ten environmental strains produce significantly elevated levels of pyocyanin compared to PAO1 (*F*_(10, 37)_ = 3.421; *P* = 0.0030; Fig 3). More specifically, we found that strain Pae110 and Pae113 produced the highest amount of pyocyanin with 14.6 (3.15) μM/OD_600_ nm and 15.72 (4.8) μM/OD_600_ nm, respectively. On the other hand, we found that strain Pae160 only produce a modest amount of pyocyanin, 3.49 (1.88) μM/OD_600_ nm, while strain Pae111 barely produced detectable amounts of pyocyanin, 0.03 (0.14) μM/OD_600_ nm. Optical density was considered for normalization of all the proceeding data.

### Exposure to antibiotic sub-MIC concentrations influences the production of pyocyanin

We investigated the potential impact of sub-inhibitory concentrations of antibiotics on virulence activity on three strains. PAO1 was selected as the reference strain and as a producer of low pyocyanin concentrations, while Pae85 was selected as a producer of high pyocyanin production and Pae112 was a producer of medium level concentrations compared to the other environmental strains we screened. The pyocyanin production of these strains were measured after exposure to 10× sub-MIC concentrations of ciprofloxacin, tobramycin, and meropenem, respectively.

**Fig 4:**
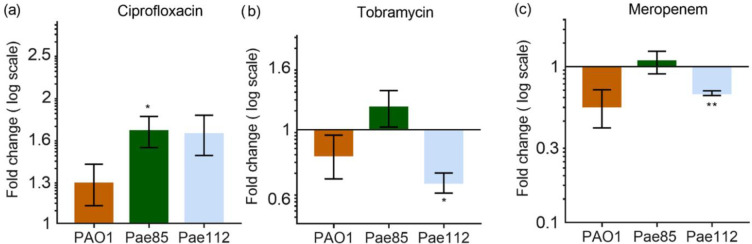
Pyocyanin quantification when strains exposed to sub-MIC antibiotic concentrations. Changes in pyocyanin levels in response to treatment of 10x sub-MIC of (a) ciprofloxacin (Cipro), (b) tobramycin (Tobra), and (c) meropenem (Mero), tested on PA01, Pae85, and Pae112. The changes in pyocyanin production are normalized using optical density (OD_600_ nm). Data from single strain analysis with means ± SEM from 3 to 4 replicates (Paired t-test, *P<0.0332, **P<0.0021).

We compared the changes of pyocyanin production affected by individual antibiotic treatment in all three strains with that of an untreated control. We then investigated the effect of one antibiotic, independently of the other two treatments for each strain using paired t-test and the data are presented in Fig 4A–4C. Individual analysis for each strain and treatment reveal that changes in pyocyanin production can be detected in Pae85 for sub-MIC concentrations of ciprofloxacin (t_(2)_ = 4.663, P = 0.043, Fig 4A and Pae112 for tobramycin (t_(2)_ = 6.354, *P* = 0.0239, Fig 4B and meropenem (t_(2)_ = 13.63, *P* = 0.0053, Fig 4C. Our results suggest that the effect of sub-inhibitory concentrations of different antibiotics can have singular effect on different bacterial strains.

### Pyocyanin affects virulence impact on host cells

**Fig 5:**
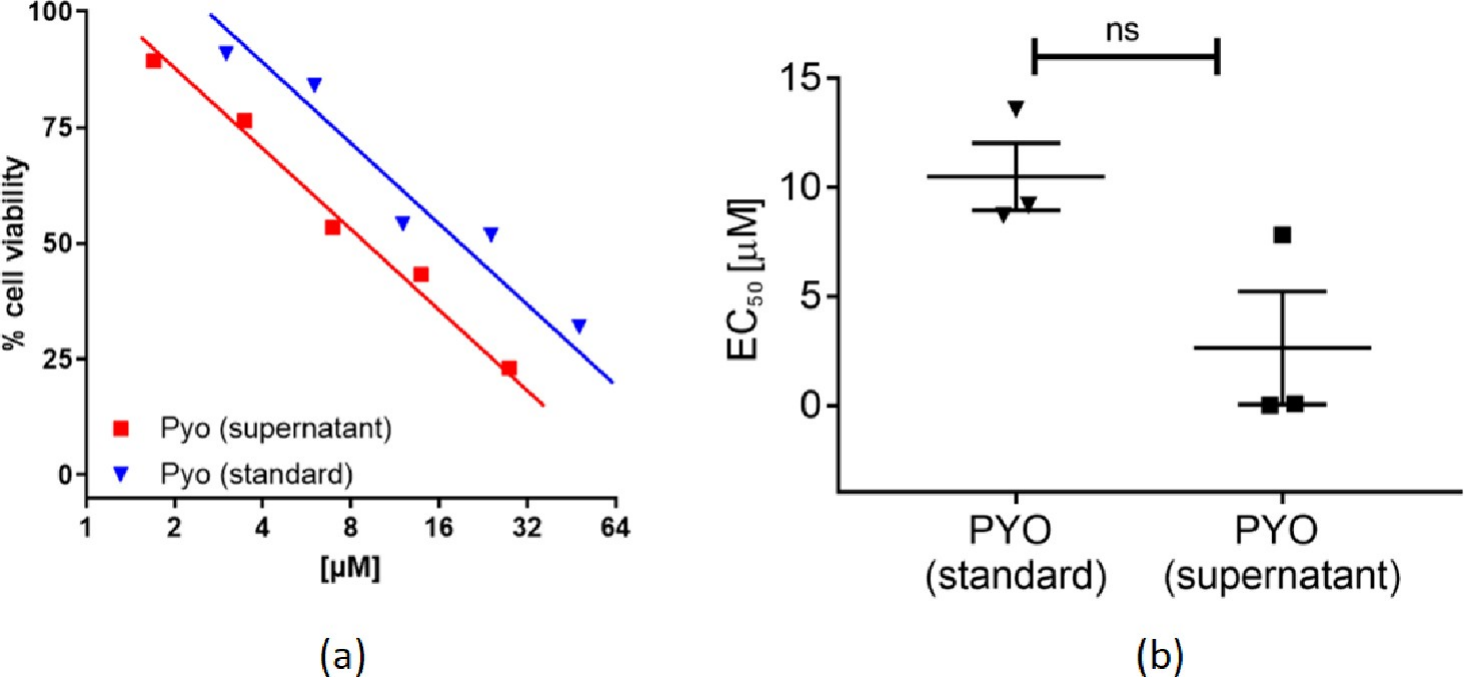
HaCaT toxicity. (a) Pyocyanin induced cell death in HaCaT cells, correlating percent cell viability with cell metabolic activity. Two pyocyanin sources were tested, standard sample where commercially available pyocyanin was used and supernatant, where PAO1 supernatant was tested and the corresponding concentrations are solely based on pyocyanin quantification present. (b) Pyocyanin concentration which gives 50% cell death (EC_50_) are plotted from three replicates with SD error bars for pyocyanin, in both standard and supernatant solutions, respectively. The average values calculated are of 10.5 and 2.6 μM, and SD of 1.5 and 2.5, respectively. Paired t-test revealed no statistical (ns) difference in toxicity between pyocyanin from standard and supernatant solutions.

The observed increase in pyocyanin production as a result of sub-MIC antibiotic exposure has a virulent impact on hosts cells. This was demonstrated by comparing the effect of a concentration series of pure pyocyanin and supernatant extracted from a PA01 culture on the viability of a monolayer of HaCaT cells. While pyocyanin extract tested for the direct effect of the molecule on virulence, the PAO1 supernatant includes other molecules in addition to pyocyanin. Interestingly, we found a similar dose-dependent toxic effect of both the PAO1 supernatant and pyocyanin pure compounds (Paired t-test; t_(2)_ = 2.12; *Ps* > 0.05; Fig 5A and 5B). This result demonstrates the virulence activity of pyocyanin on HaCaT cells and shows that pyocyanin is most likely the main component of virulence in PAO1 supernatant.

## Discussion and conclusion

Antibiotics are not always effective at killing all bacterial cells during infection. This is because not all antibiotics have the same pharmacokinetic and pharmacodynamic properties. For example, the antibiotics do not diffuse to all infection sites and bacterial biofilms can also provide a protective shield. This is important for several reasons. First, because it may lead to recurring infections, appearance of resistant or persistent bacteria and second because we do not fully understand the effects of sub-inhibitory antibiotic concentrations on bacterial pathogenesis. *P*. *aeruginosa* virulence molecules have shown to have several physiological roles [31]. However, due to challenges in quantifying these molecules, studies that report the effect of antibiotic treatment on virulence molecule secretion have been limited. In this study, we assess the effects of sub-inhibitory antibiotic concentrations on pyocyanin production through an emerging electrochemical-based technique that allows rapid and direct measurements of the virulence factor. This method allowed intensive high-throughput screenings of pyocyanin produced by *P*. *aeruginosa* strains under different antibiotics exposure. Moreover, this technique allows on-the-spot measurements of pyocyanin, without the risk of chemical degradation, concentration loss due to purification methods, or time-consuming measurements.

Our results point out that different *P*. *aeruginosa* strains produce significantly varied pyocyanin levels (Fig 3). This can translate into the ability of each strain to cause substantial toxicity upon host invasion. When three different *P*. *aeruginosa* strains were challenged with low antibiotic concentrations, the recorded levels showed different profiles (Fig 4). The differences could be attributed to their different mode of action. Ciprofloxacin (fluoroquinolone) inhibits DNA replication, tobramycin (aminoglycoside) inhibits protein synthesis and meropenem (beta-lactam) inhibits cell wall synthesis. In PAO1 ciprofloxacin caused an increase of pyocyanin production, while with tobramycin treatment, the pyocyanin levels remained unchanged. In contrast, the levels of the pyocyanin were significantly affected by this antibiotic in Pae112. Our results not only demonstrate that low concentrations of antibiotics can affect the virulence of bacterial strains but could also help identify future antimicrobial treatment in a clinical setting. For example, our data shows that low concentration of meropenem decreased pyocyanin levels in Pae112, indicating that this antibiotic could be a better choice in the clinic for the treatment of *P*. *aeruginosa* infections.

Previous studies have also reported that the accumulation of pyocyanin [32] in *P*. *aeruginosa* and that of other virulence factors [10] can enhance antibiotic tolerance. For this reason, increased pyocyanin activity could be of particular importance for resistance development especially for ciprofloxacin where resistance is present at a high level among clinical isolates [8]. Future studies may investigate the effect of *P*. *aeruginosa* virulence in correlation with resistance and tolerance profiles in response to ciprofloxacin and other antibiotics. Another aspect of change of virulence levels in presence of low concentrations of antibiotics that could be further explored is the combined effect of all three antibiotics and investigate whether the antibiotic act in synergy. This is highly important as many *P*. *aeruginosa* infections are treated simultaneously with a cocktail of antibiotics [33].

To conclude, as a proof-of-concept, we have demonstrated that sub-inhibitory concentrations of specific antibiotics impact pyocyanin production in an important bacterial model system. The main observation perceived from the present study is that if certain antibiotics do not completely eradicate the bacteria, the insufficient antibiotic concentrations induce a bacterial response in terms of virulence factor production. Future work should focus on determining why this is the case for some antibiotics and not others, how sub-inhibitory antibiotic concentrations may affect the production of other virulence factors like alginate, pyochelin and phenazines, and in which degree sub-inhibitory concentrations of antibiotics play a role in refractory infections in vivo. While further investigations are needed, we can propose that the effect of sub-MIC values on the virulence activity of bacteria is also a measure that is relevant to screen for when designing antibiotic treatments for patients. This knowledge can be another step towards more efficient patient treatment and antibiotic management.

## Supporting information

S1 Fig Effect of increasing antibiotic concentrations on the electrochemical signal. One-way ANOVA with Bonferroni multiple comparison test revealed that increasing concentrations of tobramycin (Tobra) did not affect the concentrations of pyocyanin (PAO1 supernatant) measured (*F*_(1.5,3)_ = 0.247, *P* = 0.74).
(DOCX)

S1 Table Strain number, ID and origin of all the environmental *P*. *aeruginosa* isolates [1] used in the present study. (DOCX)

S2 Table Statistical analysis. Statistical analysis data from paired t-test for the bacterial growth of the three different strains PAO1, Pae85 and Pae112, treated with three common antibiotics, ciprofloxacin (Cipro), tobramycin (Tobra) and meropenem (Mero). Paired t-test with adjusted P values from Bonferroni multiple comparison test are shown.
(DOCX)
