# Supplementary material for: Changes in toxin production of environmental Pseudomonas aeruginosa isolates exposed to sub-inhibitory concentrations of three common antibiotics
Source: PLoS One. 2021 Mar 4;16(3):e0248014. doi: 10.1371/journal.pone.0248014 (PMC7932067; doi:10.1371/journal.pone.0248014)
Supplement: S1 Fig — One-way ANOVA with Bonferroni multiple comparison test revealed that increasing concentrations of tobramycin (Tobra) did not affect the concentrations of pyocyanin (PAO1 supernatant) measured (F (1.5,3) = 0.247, P = 0.74). (DOCX) [file pone.0248014.s001.docx]

| 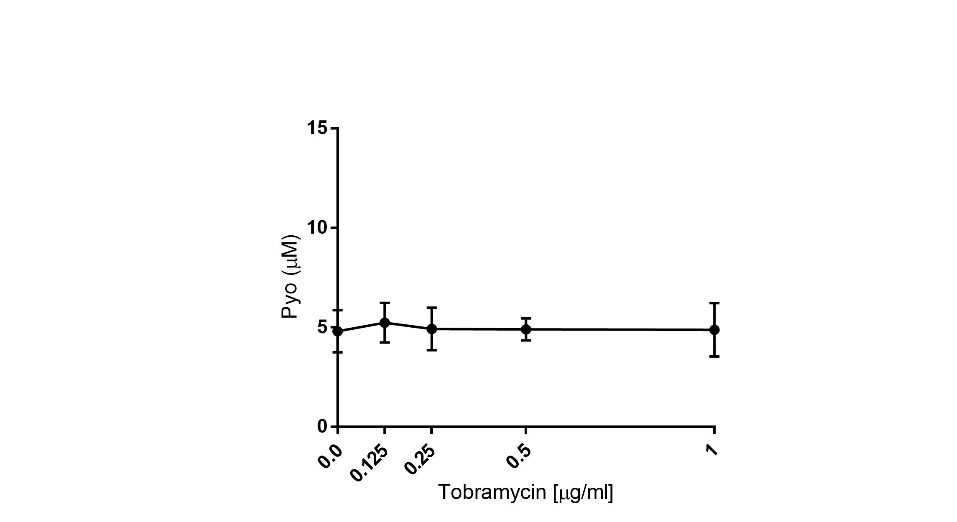 |  |
| --- | --- |

**Fig S1.** **Effect of increasing antibiotic concentrations on the electrochemical signal.** One-way ANOVA with Bonferroni multiple comparison test revealed that increasing concentrations of tobramycin (Tobra) did not affect the concentrations of pyocyanin (PAO1 supernatant) measured (*F* _(1.5,3)_ =0.247, *P*=0.74).
