# Supplementary material for: Changes in toxin production of environmental Pseudomonas aeruginosa isolates exposed to sub-inhibitory concentrations of three common antibiotics
Source: PLoS One. 2021 Mar 4;16(3):e0248014. doi: 10.1371/journal.pone.0248014 (PMC7932067; doi:10.1371/journal.pone.0248014)
Supplement: S1 Table — (DOCX) [file pone.0248014.s002.docx]

**Table S1.** **Strain number, ID and origin of all the environmental *P. aeruginosa* isolates [1] used in the present study.**

| **Strain ID** | **Origin** |
| --- | --- |
| PaeB1 | Water |
| PaeB6 | Water |
| Pae85 | Soil |
| Pae100 | Soil |
| Pae102 | Soil |
| Pae110 | Sediment |
| Pae111 | Soil |
| Pae112 | Soil |
| Pae113 | Sediment |
| Pae160 | Water |
