# Supplementary material for: Changes in toxin production of environmental Pseudomonas aeruginosa isolates exposed to sub-inhibitory concentrations of three common antibiotics
Source: PLoS One. 2021 Mar 4;16(3):e0248014. doi: 10.1371/journal.pone.0248014 (PMC7932067; doi:10.1371/journal.pone.0248014)
Supplement: S2 Table — Statistical analysis data from paired t-test for the bacterial growth of the three different strains PAO1, Pae85 and Pae112, treated with three common antibiotics, ciprofloxacin (Cipro), tobramycin (Tobra) and meropenem (Mero). Paired t-test with adjusted P values from Bonferroni multiple comparison test are shown. (DOCX) [file pone.0248014.s003.docx]

**Table S2. Statistical analysis**. Statistical analysis data from paired t-test for the bacterial growth of the three different strains PAO1, Pae85 and Pae112, treated with three common antibiotics, ciprofloxacin (Cipro), tobramycin (Tobra) and meropenem (Mero). Paired t-test with adjusted P values from Bonferroni multiple comparison test are shown

| *Comparison of negative control vs. bacterial (strain)* | *t-test, t_(df)_ value* | *P value* | *Adj-P value* |
| --- | --- | --- | --- |
| *Cipro (PAO1)* | 1.873 _(3)_ | 0.1577 | 0.4732 |
| *Tobra (PAO1)* | 0.3907_(3)_ | 0.7221 | >0.9999 |
| *Mero (PAO1)* | 2.152 _(3)_ | 0.1205 | 0.3615 |
| *Cipro (Pae85)* | 2.077 _(2)_ | 0.1735 | 0.5201 |
| *Tobra (Pae85)* | 1.003 _(2)_ | 0.4214 | >0.9999 |
| *Mero (Pae85)* | 0.6152 _(2)_ | 0.6001 | >0.9999 |
| *Cipro (Pae112)* | 3.814 _(2)_ | 0.0624 | 0.1871 |
| *Tobra (Pae112)* | 0.755 _(2)_ | 0.529 | >0.9999 |
| *Mero (Pae112)* | 0.748 _(2)_ | 0.532 | >0.9999 |

References

1. S. D. Shrestha, D. S. Guttman, and G. G. Perron, “Draft genome sequences of 10 environmental Pseudomonas aeruginosa strains isolated from soils, sediments, and waters,” Genome Announc., vol. 5, no. 34, Aug. 2017.
